# Synthesis of piperidine and pyrrolidine derivatives by electroreductive cyclization of imine with terminal dihaloalkanes in a flow microreactor

**DOI:** 10.3762/bjoc.18.39

**Published:** 2022-03-29

**Authors:** Yuki Naito, Naoki Shida, Mahito Atobe

**Affiliations:** 1 Graduate School of Science and Engineering, Yokohama National University, Yokohama, Kanagawa 240-8501, Japan

**Keywords:** electrochemical synthesis, electrocyclization, flow microreactor, heterocyclic amines, imine

## Abstract

We have successfully synthesized piperidine and pyrrolidine derivatives by electroreductive cyclization using readily available imine and terminal dihaloalkanes in a flow microreactor. Reduction of the substrate imine on the cathode proceeded efficiently due to the large specific surface area of the microreactor. This method provided target compounds in good yields compared to a conventional batch-type reaction. Furthermore, piperidine and pyrrolidine derivatives could be obtained on preparative scale by continuous electrolysis for approximately 1 hour.

## Introduction

**Figure 1:**
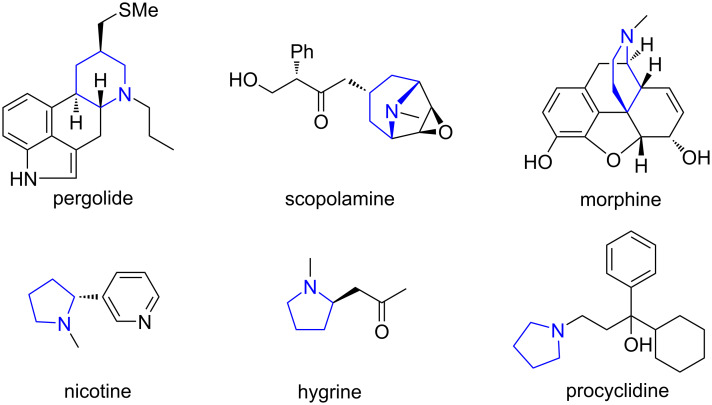
Piperidine and pyrrolidine rings in biologically active compounds.

Heterocycles are a very important class of compounds and make up more than half of all known organic chemicals [1]. Among them, heterocyclic amines, particularly pyrrolidine and piperidine derivatives, have attracted considerable attention because these are important structural motifs in a wide variety of applications including pharmaceuticals, natural products, and biologically active compounds such as pergolide, scopolamine, morphine, nicotine, hygrine, and procyclidine (Figure 1) [2–4]. Therefore, a considerable number of synthetic approaches to pyrrolidines and piperidines have been investigated [5–13].

**Scheme 1:**
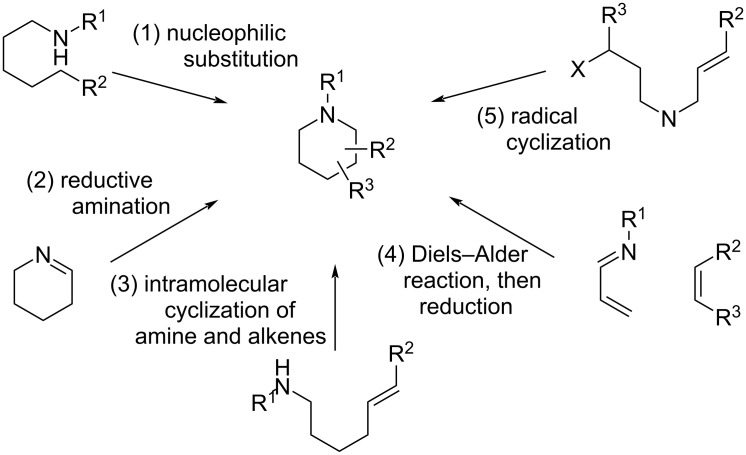
Conventional synthetic routes for piperidine derivatives.

Conventional synthetic methods for piperidine derivatives include nucleophilic substitution (route (1) in Scheme 1), reductive amination (route (2)), intramolecular cyclization of amines and alkenes (route (3)), the Diels–Alder reaction and subsequent reduction (route (4)), and the radical cyclization reaction (route (5)). However, these methods involve the use of toxic acids, bases, or transition metal catalysts, and typically require elevated temperatures [14–20]. In addition, very recently, Molander and co-workers have developed a photoredox-mediated radical/polar crossover process which realizes for the construction of medium-sized saturated nitrogen heterocycles [21]. Although the process enables the rapid construction of saturated nitrogen heterocycles from acyclic precursors, it requires homogeneous precious transition metal complexes as photocatalysts.

**Scheme 2:**
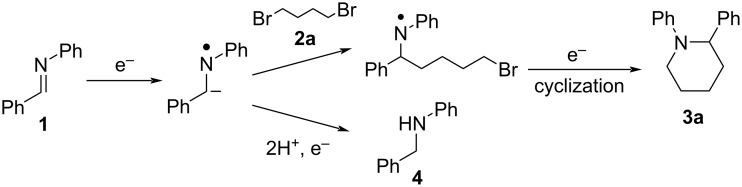
Synthesis of 1,2-diphenylpiperidine (**3a**) by the electroreductive cyclization mechanism.

On the other hand, organic electrosynthetic reactions, which are driven by direct electron transfer to and from the electrodes, can produce highly reactive species under ambient conditions without the use of harmful and precious chemicals. Therefore, electrosynthesis has been actively researched in recent years as a green and sustainable synthetic method in the face of increasingly stringent environmental and economic constraints. In this context, several groups have demonstrated the electrochemical synthesis of piperidine and pyrrolidine derivatives by anodic oxidation [22–26]. In contrast, there has been only one report on the electroreductive synthesis of piperidine derivatives: namely Degrand and co-workers demonstrated electroreductive cyclization using imines and terminal dihaloalkanes to provide piperidine derivatives (Scheme 2) [27].

In this reaction, a stable radical anion is produced from the starting imine **1** in the first reduction step. The nucleophilic attack of the radical anion on the terminal dihaloalkane **2a** is the second step, which forms a radical. These sequential steps are followed by a further one-electron reduction at the cathode to provide an anion intermediate. The last step is cyclization of the mono-substituted anion to provide the cyclization product **3a**. In addition, the hydromonomeric product **4** was also formed as a byproduct. Although Degrand et al. [27] could successfully obtain the piperidine derivatives by this reductive cyclization; a toxic mercury pool cathode was used in their demonstration. Therefore, it is desirable to conduct the reductive cyclizations without the use of a mercury cathode, and the development of a simple, green, and efficient method for the electrochemical synthesis of heterocyclic amines is an important research target.

The electrochemical flow microreactor has recently attracted attention as an excellent alternative tool to conventional batch-type electrochemical reactors [28–32]. The potential advantages of electrochemical flow microreactors over conventional batch-type reactors are a large surface-to-volume ratio, precise residence time, extremely fast molecular diffusion, and expelling the reaction product to avoid over-oxidation or over-reduction. We have previously reported the electrochemical carboxylation of several imines in a flow microreactor to afford the corresponding α-amino acids in good to moderate yields [33–35]. The key features of this method are the effective cathodic reduction of imines and their rapid use for the subsequent reactions in a microflow system. Successful preliminary results prompted us to perform the electroreductive cyclization of an imine with terminal dihaloalkanes to afford heterocyclic amines in a flow microreactor because the reaction involves cathodic reduction of the imine and its rapid use for the subsequent reaction with the terminal dihaloalkanes.

In this work, we demonstrate the electroreductive cyclization of an imine with terminal dihaloalkanes in a flow microreactor to establish a facile, green, and efficient method for the synthesis of heterocyclic amines such as pyrrolidine and piperidine derivatives.

## Results and Discussion

**Figure 2:**
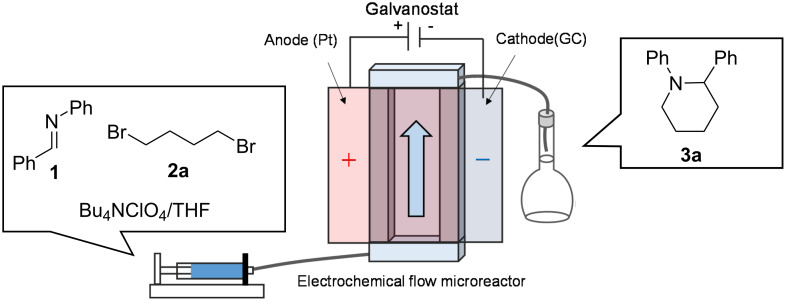
Schematic diagram of the electroreductive cyclization for the synthesis of 1,2-diphenylpiperidine (**3a**) in an electrochemical flow microreactor. Adapted with permission from ref. [33]. Copyright 2021 American Chemical Society. This content is not subject to CC BY 4.0.

As a model reaction, the electroreductive cyclization of benzylideneaniline with 1,4-dibromobutane to provide 1,2-diphenylpiperidine was selected. The microreactor fabricated for the model reaction had a simple geometry with the cathode and anode directly facing each other at a distance of several tens of micrometers (Figure 2). The electrolyte containing benzylideneaniline (**1**) and 1,4-dibromobutane (**2a**) was pumped into the gap between the two electrodes and subjected to the electrolytic reaction. Tetrahydrofuran (THF), which is easily oxidized, was employed as the electrolytic solvent, so the reaction at the anode (counter electrode) is thought to be preferentially caused by the oxidative decomposition of THF, and the re-oxidation of the cathodic reaction products at the anode would be suppressed.

On the other hand, the cathode material is an important factor in selecting the course of the cathodic reaction and to control the efficiency of the reaction. To select a suitable cathode material for this model reaction, we first investigated the effect of the cathode material on the yield of **3a** using three different cathode materials: platinum (Pt), glassy carbon (GC), and silver (Ag). As shown in Table 1, the yield of **3a** was higher using GC than that of the other cathode materials. The yield of the hydromonomeric product **4** was also highest when GC was used. Both **3a** and **4** are products obtained through the reduction of imine **1**, which suggests that the GC cathode is effective for the reduction of imine **1**. In sharp contrast, the yields of both **3a** and **4** were very low when a Ag cathode was used. A Ag cathode has electrocatalytic activity for the reduction of organic halides [35–36]; therefore, 1,4-dibromobutane (**2a**) was more easily reduced than **1** at the Ag cathode, which resulted in the recovery of a large amount of unreacted **1**. Linear sweep voltammetry (LSV) experiments revealed that the reduction of **2a** occurred at a lower potential than the reduction of **1** when the Ag cathode was used (Supporting Information File 1, Figure S7). Furthermore, LSV experiments with various cathodes showed that the reduction of **2a** was significantly dependent on the cathode material, and their overpotentials were larger in the order of Ag < Pt < GC (Supporting Information File 1, Figures S5–S7). Therefore, GC prioritized the reduction of **1**, even in the presence of **2a**, to produce **3a** and **4** efficiently. In the following experiments, GC was used as the cathode material for this reaction.

**Table 1:** Effect of the cathode material on the reduction products, **3a** and **4**^a^.

Entry	Cathode material	Yield of **3a** (%)^b^	Yield of **4** (%)^b^

1	Pt	21	27
2	GC	36	53
3	Ag	2	2

^a^Experimental conditions: anode, Pt plate; electricity, 2.15 F mol^−1^; current density, 12.7 mA cm^−2^; electrode distance, 40 μm; solvent, THF; substrate, 0.06 M benzylideneaniline (**1**) and 0.06 M 1,4-dibromobutane (**2a**); supporting electrolyte, 0.14 M *n-*Bu_4_N∙ClO_4_; flow rate, 11 mL h^−1^ (residence time, 3.9 s). ^b^Determined by HPLC.

The effect of the amount of **2a** addition on the yield of **3a** was then investigated. Table 2 shows that the yield of **3a** increased as the amount of **2a** was increased, and reached a maximum at 2 equiv. The yield of **3a** began to decrease with the addition of more **2a**. This decrease in the yield of **3a** can be attributed to the competition of the cathodic reduction of **2a** caused by the increase in the amount of **2a** addition. On the other hand, as shown in Scheme 2, the desired product **3a** is produced by the reaction of **2a** with the radical anion species of the substrate imine **1**, while the hydromonomeric product **4** is produced by the reaction of protons with the radical anion species of **1**. Therefore, the yield of **4** consistently decreased with the increase in the amount of **2a** addition.

**Table 2:** Effect of the amount of 1,4-dibromobutane (**2a**) addition on the yield of the reduction products **3a** and **4**^a^.

Entry	1,4-Dibromobutane (equiv)	Yield of **3a** (%)^b^	Yield of **4** (%)^b^

1	1.0	36	53
2	1.5	45	40
3	2.0	47	33
4	3.0	28	29
5	5.0	26	24

^a^Experimental conditions: cathode, GC plate; anode, Pt plate; electricity, 2.15 F mol^−1^; current density, 12.7 mA cm^−2^; electrode distance, 40 μm; solvent, THF; substrate, 0.06 M benzylideneaniline (**1**) and 1,4-dibromobutane (**2a**); supporting electrolyte, 0.14 M *n-*Bu_4_N∙ClO_4_; flow rate, 11 mL h^−1^ (residence time, 3.9 s). ^b^Determined by HPLC.

In the model process in an electrochemical flow microreactor, the desired reaction (the reaction of radical anion of imine **1** with **2a** to afford **3a**) at the cathode may be interfered with by the protons generated by the oxidation of the THF solvent at anode, leading to increased formation of **4** because the distance between the electrodes of the reactor is much shorter than that of conventional batch-type reactors. Therefore, the electrode distance is also an important factor that must be investigated to obtain the desired product **3a** in a high yield. Table 3 shows the effect of the electrode distance on the yield of the reduction products **3a** and **4**. From these results, the optimal electrode distance was determined to be 40 µm (Table 3, entry 2). When the electrode distance was increased to 80 µm, the yield of **3a** decreased (Table 3, entry 1). This may be ascribed to the decrease in the surface-to-volume ratio, which makes it difficult for imine **1** to reach the cathode during the residence time. On the other hand, when the electrode distance was decreased to 20 µm, not only the yield of **3a**, but also the selectivity toward **3a**/**4** decreased (Table 3, entry 3). As mentioned above, this is probably because the protons generated by the oxidation of the THF solvent at anode could easily meet the radical anions of imine **1** generated at cathode due to the very short interelectrode distance.

**Table 3:** Effect of the electrode distance on the yield of the reduction products **3a** and **4**^a^.

Entry	Electrode distance/µm (residence time/s)	Yield of **3a** (%)^b^	Yield of **4** (%)^b^	Selectivity **3a**/**4**

1	80 (7.9)	35	26	1.35
2	40 (3.9)	47	33	1.42
3	20 (2.0)	21	27	0.78

^a^Experimental conditions: cathode, GC plate; anode, Pt plate; solvent, electricity, 2.15 F mol^−1^; current density, 12.7 mA cm^−2^; solvent, THF; substrate, 0.06 M benzylideneaniline (**1**) and 0.12 M 1,4-dibromobutane (**2a**); supporting electrolyte, 0.14 M *n-*Bu_4_N∙ClO_4_; flow rate, 11 mL h^−1^. ^b^Determined by HPLC.

In the electrochemical reaction, the electrons themselves act as reagents, so electricity affects the degree of reaction progress. In addition, excessive electricity may also cause undesired electrochemical reactions, and it is thus extremely important to estimate the optimal electricity for the desired reaction. For constant-current electrolysis in a flow microreactor with fixed channel dimensions, the electricity can be controlled by changing the current density or flow rate. As shown in entries 1 and 2 of Table 4, the yield of **3a** increased with the electricity (caused by an increase in the current density) and reached a maximum value at 2.15 F mol^−1^. The theoretical electricity required for the generation of **3a** is 2 F mol^−1^; however, 2 F mol^−1^ of electricity was probably insufficient to fully convert the substrate imine **1** introduced into the reactor. On the other hand, as shown in Table 4, entries 3–5, the yield of **3a** decreased slightly when the electricity was increased from 2.15 F mol^−1^ and above, and in contrast, there was an increasing trend in the yield of **4**. This may be ascribed to an increase in the proton supply resulting from THF oxidation at the anode due to an increase in the electricity, which promoted the formation of **4** by the reaction between protons and the radical anions of **1**. As shown in Table 4, entry 6, the yield of **3a** decreased when the electricity was fixed at 2 F mol^−1^ by increasing the flow rate over the conditions in Table 4, entry 2. On the other hand, as shown in Table 4, entry 7, the yield of **3a** was also decreased compared to entry 2 when the electricity was increased to 3 F mol^−1^ by decreasing the flow rate.

**Table 4:** Effect of electricity on the reduction products **3a** and **4**^a^.

Entry	Electricity /F mol^–1^	Current density /mA cm^–2^	Flow rate /mL h^–1^ (residence time/s)	Yield of **3a** (%)^b^	Yield of **4** (%)^b^

1	2.0	11.8	11 (3.9)	33	21
2	2.15	12.7	11 (3.9)	47	33
3	2.3	13.6	11 (3.9)	37	37
4	2.5	14.0	11 (3.9)	34	29
5	3.0	17.7	11 (3.9)	35	46
6	2.0	12.7	12 (3.6)	23	13
7	3.0	12.7	8 (5.4)	31	26

^a^Experimental conditions: cathode, GC plate; anode, Pt plate; solvent, THF; electrode distance, 40 μm; substrate, 0.06 M benzylideneaniline (**1**) and 0.12 M 1,4-dibromobutane (**2a**); supporting electrolyte, 0.14 M *n-*Bu_4_N∙ClO_4_. ^b^Determined by HPLC.

The rate of the nucleophilic reaction between the radical anion intermediate generated reductively from **1** and **2a** would be influenced by the cation size of the supporting electrolyte used because this leads to different ion-pair interactions with the radical anion intermediate. To confirm this conjecture, we carried out the model reaction using perchlorate salts consisting of cations of different sizes. As shown in entry 2 of Table 5, when Et_4_N∙ClO_4_ was used as the supporting electrolyte, the electrolysis reaction could not be carried out because Et_4_N∙ClO_4_ did not dissolve in the THF solution. On the other hand, *n-*Hex_4_N∙ClO_4_ dissolved easily in THF solution and the electrolysis reaction proceeded smoothly. However, the yield of the desired product **3a** was slightly lower than that using *n-*Bu_4_N∙ClO_4_. Therefore, *n-*Bu_4_N∙ClO_4_ is the most suitable supporting electrolyte for the model reaction among the tested electrolytes.

**Table 5:** Effect of the supporting electrolyte on the yield of the reduction products **3a** and **4**^a^.

Entry	Supporting electrolyte	Yield of **3a** (%)^b^	Yield of **4** (%)^b^

1	*n-*Bu_4_N∙ClO_4_	47	33
2	Et_4_N∙ClO_4_^c^	–	–
3	*n-*Hex_4_N∙ClO_4_	39	18

^a^Experimental conditions: cathode, GC plate; anode, Pt plate; electricity, 2.15 F mol^−1^; current density, 12.7 mA cm^−2^; electrode distance, 40 μm; solvent, THF; substrate, 0.06 M benzylideneaniline (**1**) and 1,4-dibromobutane (**2a**); concentration of supporting electrolyte, 0.14 M; flow rate, 11 mL h^−1^ (residence time, 3.9 s). ^b^Determined by HPLC. ^c^Et_4_N∙ClO_4_ did not dissolve in THF solution.

In these investigations, the yield of the desired product **3a** was improved to 47% (entry 1 of Table 5); however, this was still not sufficient. To further improve the yield, it would be effective to suppress the competing formation of **4**. To meet this challenge, we attempted to add various bases to the electrolyte to capture the protons in the reaction system. Table 6 shows that the yields of **3a** and **4** were strongly influenced by the type of base added. When 1,8-diazabicyclo[5,4,0]-7-undecene (DBU), which has the strongest basicity among these bases, was used, the yield of **3a** increased significantly, while the formation of **4** was suppressed. In particular, when more than 1.0 equiv of DBU was added, the yield of **3a** reached almost 80%.

**Table 6:** Effect of the type of added base on the yield of the reduction products **3a** and **4**^a^.

Entry	Base		Yield of **3a** (%)^c^	Yield of **4** (%)^c^

	Type (p*K*_a_ of conjugated acid of base^b^)	equiv		

1	pyridine (5.33)	0.5	33	19
2	2,6-lutidine (6.7)	0.5	38	30
3	piperidine (11.1)	0.5	31	28
4	DBU (12.0)	0.5	61	25
5	DBU (12.0)	1.0	78	11
6	DBU (12.0)	1.5	77	17

^a^Experimental conditions: cathode, GC plate; anode, Pt plate; electricity, 2.15 F mol^−1^; current density, 12.7 mA cm^−2^; electrode distance, 40 μm; solvent, THF; substrate, 0.06 M benzylideneaniline (**1**) and 0.12 M 1,4-dibromobutane (**2a**); base added, 0.06 M DBU; supporting electrolyte, 0.14 M *n-*Bu_4_N∙ClO_4_; flow rate, 11 mL h^−1^ (residence time, 3.9 s). ^b^Literature data according to ref. [37–41]. ^c^Determined by HPLC.

The reactivity of the radical anions of imine **1** with the terminal dihaloalkane is also considered to be an important factor in the formation of the desired product **3a**. Therefore, a model reaction was conducted using dihaloalkanes with different types of terminal halogens (Cl, Br, and I) (Table 7). When the terminal halogen of the dihaloalkane was changed from Br to Cl, the yield of **3a** decreased significantly (Table 7, entry 2). This is probably due to the poor leaving ability of Cl and its low reactivity with the radical anion species generated from imine **1**. This is supported by the increase in the formation of the competing **4**. On the other hand, the yield of **3a** decreased even when the terminal halogen of the dihaloalkane was changed to iodine (Table 7, entry 3). LSV measurements showed that the reduction potential of 1,4-diiodobutane (**2c**) was almost the same as that of the substrate imine **1** (Supporting Information File 1, Figure S12); therefore, the competition between the reduction of **2c** and that of the imine **1** may have caused the low yield of **3a**. A large amount (50%) of unreacted **1** was recovered in this case. Therefore, it can be stated that Br is appropriate as the terminal halogen of the dihaloalkane in this cyclization reaction.

**Table 7:** Yields of **3a** and **4** in the model reductive cyclization using dihaloalkanes with different types of terminal halogens^a^.

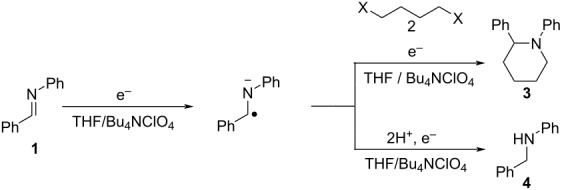			

Entry	Type of X	**3a**^b^ (%)	**4**^b^ (%)

1	Br (**2a**)	78	11
2	Cl (**2b**)	6	72
3	I (**2c**)	14	13

^a^Experimental conditions: cathode, GC plate; anode, Pt plate; electricity, 2.15 F mol^−1^; current density, 12.7 mA cm^−2^; electrode distance, 40 μm; solvent, THF; substrate, 0.06 M benzylideneaniline (**1**) and 0.12 M 1,4-dihaloalkane (**2a**, **2b**, or **2c**); base added, 0.06 M DBU; supporting electrolyte, 0.14 M *n-*Bu_4_N∙ClO_4_; flow rate, 11 mL h^−1^ (residence time, 3.9 s). ^b^Determined by HPLC.

A model electroreductive cyclization was subsequently conducted in a conventional batch-type reactor and a flow microreactor with the same electrolytic parameters (Table 8). The yield of **3a** was much higher in the electrochemical flow microreactor than in the batch-type reactor. In the batch system, the concentration of substrate imine **1** in the electrolyte decreases as the electrolysis progresses, which makes it difficult for the reaction to proceed. Therefore, even after passing more than the theoretical amount of electricity, a considerable amount (28%) of imine **1** remained. In contrast, in the flow system, the electrolyte containing a predetermined amount of imine **1** is always supplied from the reactor inlet, and the relatively fast flow in the reactor provides a good supply of imine **1** to the working electrode (cathode), so the steady state may be maintained in the flow microreactor. Such an ideal environment for the reduction of imine **1** is considered to result in the high yield of **3a**.

**Table 8:** Effect of reactor type on the yield of the reduction products **3a** and **4**^a^.

Entry	Reactor type	Electrode distance/µm	Yield of **3a** (%)^b^	Yield of **4** (%)^b^

1	batch	2 cm	45	25
2	flow	40 µm	77	20

^a^Experimental conditions: cathode, GC plate; anode, Pt plate; electricity, 2.15 F mol^−1^; current density, 12.7 mA cm^−2^; solvent, THF (10 mL each were used for batch and flow experiments.); substrate, 0.06 M benzylideneaniline (**1**) and 0.12 M 1,4-dibromobutane (**2a**); base added, 0.06 M DBU; supporting electrolyte, 0.14 M *n-*Bu_4_N∙ClO_4_; flow rate, 11 mL h^−1^ (residence time, 3.9 s). ^b^Determined by HPLC.

**Figure 3:**
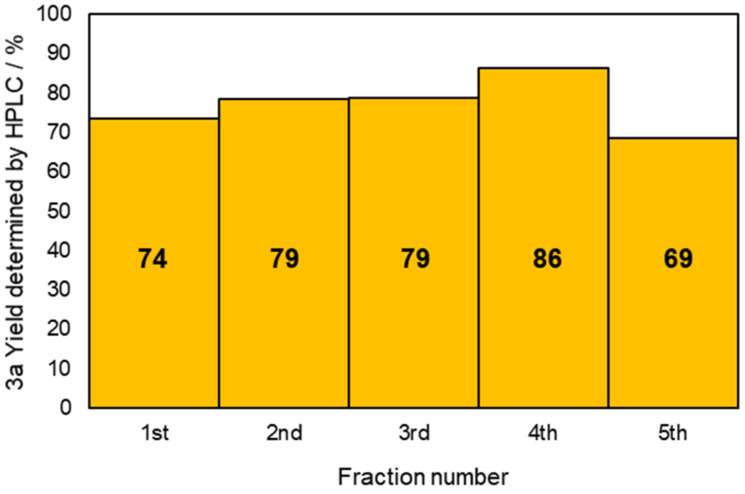
Yield of **3a** for each fraction sample in the continuous flow reductive cyclization.

To confirm that the reaction environment of the flow system was in the steady state, the electrolyzed solution was collected from the reactor outlet at regular intervals and the yield of **3a** for each fraction was determined (Figure 3). Continuous flow electrolysis could be performed without any problem at least until the fifth fraction collection. The yields of the fractions were almost the same, and the average yield of the five fractions was 77%. In addition, no precipitates were observed on the electrodes after the electrolysis, which suggests that a stable yield could be maintained. Therefore, it can be stated that the reaction environment of the flow system was in the steady state.

Experimental conditions for the collection of each fraction sample: cathode, GC plate; anode, Pt plate; electricity, 2.15 F mol^−1^; current density, 12.7 mA cm^−2^; solvent, THF; substrate, 0.06 M benzylideneaniline (**1**) and 0.12 M 1,4-dibromobutane (**2a**); base added, 0.06M DBU; supporting electrolyte, 0.14 M *n-*Bu_4_N∙ClO_4_; flow rate, 11 mL h^−1^ (residence time, 3.9 s); collection volume and time for each fraction, 2 mL, 10 min 55 s. The yield of **3a** was determined by HPLC.

After mixing the five fraction samples, we attempted to isolate **3a** from the mixture, and obtained 78.4 mg (55% isolated yield) of **3a** (entry 1 of Table 9). The desired piperidine derivative could be obtained on a scale of several tens of milligrams in approximately 1 hour of continuous electrolysis; therefore, we then attempted to synthesize pyrrolidine and azetidine derivatives (**3b** and **3c**) using the same procedure (entries 2 and 3 of Table 8). **3b** was obtained from imine **1** and dihaloalkane **2d** with a good isolated yield (57%, 75.8 mg). However, the azetidine derivative **3c** was not obtained at all by the reductive cyclization of **1** and **2e**. LSV experiments revealed that the reduction of **2e** occurred at a slightly lower potential than the reduction of **1** (Supporting Information File 1, Figure S14). Therefore, in this case, the competitive reduction of **2e** would inhibit the desired cyclization reaction via the reduction of **1**.

**Table 9:** Isolated yield of heterocyclic amines (**3a**–**c**) obtained by the reductive cyclization of imine **1** with various dihaloalkanes (**2a**, **2d**, and **2e**)^a^.

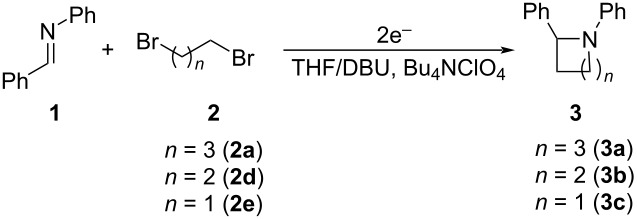		

Entry	Terminal dihaloalkane **2**	Isolated yield of **3** (%)

1	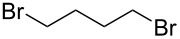 **2a**	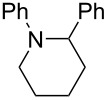 **3a** 55%
2	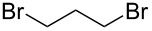 **2d**	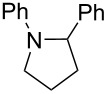 **3b** 57%
3	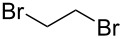 **2e**	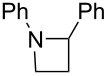 **3c** n.d.

^a^Experimental conditions: cathode, GC plate; anode, Pt plate; electricity, 2.15 F mol^−1^; current density, 12.7 mA cm^−2^; electrode distance, 40 μm; solvent, THF; substrate, 0.06 M benzylideneaniline (**1**) and 0.12 M terminal dihaloalkane (**2a**, **2d**, or **2e**); base added, 0.06 M DBU; supporting electrolyte, 0.14 M *n-*Bu_4_N∙ClO_4_; flow rate, 11 mL h^−1^ (residence time, 3.9 s); collection volume and time for the reaction solution, 10 mL, 54 min 35 s.

## Conclusion

We have demonstrated a facile, green, and efficient method for the synthesis of heterocyclic amines by electroreductive cyclization using a flow microreactor. This method not only allows the synthesis of pyrrolidine and piperidine derivatives from readily available compounds in a single step, but also has the advantage of eliminating the use of expensive or toxic reagents. In addition to optimization of the general parameters for the electrolytic reaction, the effect of base addition was also investigated, and was determined to suppress the formation of the hydromonomeric product, which is a main byproduct. Among the various bases, DBU suppressed the formation of byproducts and the desired cyclization products such as piperidine and pyrrolidine derivatives were obtained in good yields. Moreover, in the electrochemical flow microreactor, the yield of the desired product was much higher than in the batch reactor. These findings provide new insights into the synthetic chemistry of heterocyclic amines.

## Experimental

### General considerations

All chemicals were used without further purification. **1**, **2a**–**e,** and **4** were purchased from commercial sources. **3a, 3b**, and **3c** were synthesized according to reported procedures (see Supporting Information File 1). A silicon oil bath was used as a heat source for the synthesis of **3a**. Flow electrolysis was conducted with an in-house-built electrochemical flow microreactor. The substrate solution was introduced to the microreactor by a syringe pump (KDS100, KdScientific Muromachi Kikai) during the electrosynthesis. Electroreductive cyclization was conducted using a potentiogalvanostat (HABF-501A, Hokuto Denko). High-performance liquid chromatography (HPLC) analysis for **3a** and **4** was performed with a column (Mightysil RP-18 GP II 250-4.6 (5 µm), Cica) using a mixture of H_2_O/MeCN/H_3_PO_4_ (60/40/0.1%) as a mobile phase. All chromatograms were recorded using an LC workstation (LabSolutions DB, Shimadzu). Helium gas was used as a carrier gas for the gas chromatography/mass spectrometry (GC–MS) analyses. ^1^H nuclear magnetic resonance (NMR) spectra were recorded on a spectrometer (DRX-500, Bruker; 500 MHz) using tetramethylsilane (TMS) as an internal standard with the solvent resonance (CDCl_3_: δ 7.26). The chemical shifts for ^1^H NMR spectra are given in δ (ppm) relative to the TMS internal standard. Multiplicities are abbreviated as singlet (s), doublet (d), triplet (t), doublet of triplet (dt), and multiplet (m). Linear sweep voltammetry (LSV) was performed using an electrochemical analyzer (630c, ALS/H CH Instruments).

#### Fabrication of the electrochemical flow microreactor

The electrochemical flow microreactor was constructed with a platinum plate (3 × 3 cm) and cathode plate (3 × 3 cm) (Supporting Information File 1, Figure S1). A spacer (double faced adhesive type with thicknesses of 20, 40, or 80 μm) was used to leave a rectangular channel exposed (1 × 3 cm), and the two electrodes were simply sandwiched together. After connecting Teflon tubings to the inlets and outlet, the reactor was sealed with epoxy resin (Supporting Information File 1, Figure S2). Thus, the dimensions of the flow channel in the reactor are 1 cm width and 3 cm length, and the channel height corresponds to the thickness of the spacer (20, 40, or 80 μm).

#### Procedure for the electroreductive cyclization using an electrochemical flow microreactor

The flow microreactor system for the model synthetic reaction was fabricated as illustrated in Supporting Information File 1, Figure S1. The electroreductive cyclization reaction was conducted by introduction of a solution (*n-*Bu_4_N∙ClO_4_ or *n-*Hex_4_N∙ClO_4_ in THF) containing imine **1**, terminal dihaloalkane **2**, and a base into the electrochemical flow microreactor from a syringe pump. Imine **1** is reduced at the cathode in the flow microreactor and then the generated radical anions of **1** react with the terminal dihaloalkane to produce the corresponding heterocyclic compounds. The THF solvent is oxidized at the anode to generate protons. After electrolysis, 1 mL of the reaction solution was collected from the outlet of the reactor, diluted 5 times with THF, and analyzed using HPLC.

#### Procedure for the electroreductive cyclization using a batch-type reactor

10 mL of a solution (*n-*Bu_4_N∙ClO_4_ in THF) containing 0.06 M benzylideneaniline (**1**), 0.12 M 1,4-dibromobutane (**2a**), and 0.06 M DBU was prepared. This solution was then added to an undivided cell equipped with a working electrode (GC plate, 1 × 3 cm) and a counter electrode (Pt plate, 1 × 3 cm). The distance between the two electrodes was set to ca. 2 cm. Constant current (12.7 mA cm^−2^) was subsequently applied for the electrolysis reaction. After passage of the electricity (2.15 F mol^−1^), the electrolyzed solution was diluted 5 times with THF and analyzed using HPLC.

## Supporting Information

File 1 Detailed experimental procedures, analytical data, and supplementary figures, and photographs.
