# Supplementary material for: Synthesis of piperidine and pyrrolidine derivatives by electroreductive cyclization of imine with terminal dihaloalkanes in a flow microreactor
Source: Beilstein J Org Chem. 2022 Mar 29;18:350–9. doi: 10.3762/bjoc.18.39 (PMC8978912; doi:10.3762/bjoc.18.39)
Supplement: File 1 — Detailed experimental procedures, analytical data, and supplementary figures, and photographs. [file Beilstein_J_Org_Chem-18-350-s001.pdf]

BEILSTEIN JOURNAL OF ORGANIC CHEMISTRY

Supporting Information

for

Synthesis of piperidine and pyrrolidine derivatives by electroreductive cyclization of imine with terminal dihaloalkanes in a flow microreactor

Yuki Naito, Naoki Shida and Mahito Atobe

Beilstein J. Org. Chem. **2022,** *18,* 350–359. doi:10.3762/bjoc.18.39

Detailed experimental procedures, analytical data, and supplementary figures, and photographs

License and Terms: This is a supporting information file under the terms of the Creative Commons Attribution License (https://creativecommons.org/licenses/by/4.0). Please note that the reuse, redistribution and reproduction in particular requires that the author(s) and source are credited and that individual graphics may be subject to special legal provisions.
The license is subject to the *Beilstein Journal of Organic Chemistry* terms and conditions: (https://www.beilstein-journals.org/bjoc/terms)

Contents

Synthesis	S2
Fabrication of electrochemical flow microreactor	S4
Procedure for electroreductive cyclization in an electrochemical flow microreactor	S5
Procedure for electroreductive cyclization in a batch-type reactor	S6
Linear sweep voltammetry	S7
HPLC analysis	S13
^{1}H NMR spectra	S15
Supporting reference	S18

1. Synthesis

1-1. Procedure for the synthesis of 3a as an authentic sample for HPLC analysis.

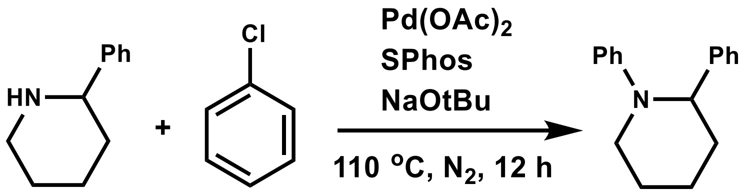

1,2-Diphenylpiperidine (**3a**) was synthesized according to a previously reported procedure.[1] A flask with a magnetic stirring bar was charged with chlorobenzene (2.1 mmol, 236 mg), 2-phenylpiperidine (2 mmol, 323 mg), $Pd(OAc)_2$ (0.02 mmol, 5 mg), SPhos (0.04 mmol, 16 mg), and powdered NaO*t*-Bu (2.4 mmol, 231 mg). The flask was transferred to a preheated oil bath (110 °C). After 12 h, the reaction mixture was cooled and dissolved in a CH_2Cl_2/H_2O mixture (1:1). The organic phase was separated and the solvent was evaporated in vacuo. The residue was purified by column chromatography using silica gel (hexane/Et_2O 95:5) to afford the product **3a**.

1,2-Diphenylpiperidine (**3a**):[2] yellow liquid, 1.6 mg, 13% yield. ^1H NMR (500 MHz; $CDCl_3$): δ 7.27-7.20 (m, 4H), 7.16-7.10 (m, 3H), 6.89 (m, 2H), 6.75 (t, *J* = 7.2 Hz, 1H), 4.50 (dd, *J* = 6.9, 4.6 Hz, 1H), 3.42-3.38 (m, 1H), 3.28-3.23 (m, 1H), 2.02-1.51 (m, 6H).

1-2. Procedure for the preparative scale synthesis of 3a-c by electroreductive cyclization using an electrochemical flow microreactor.

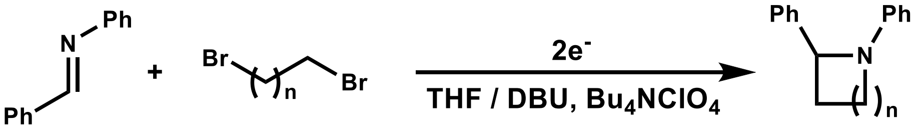

1 2 n = 3 (2a) 3 n = 3 (3a)
n = 2 (2d) n = 2 (3b)
n = 1 (2e) n = 1 (3c)

Heterocyclic amines **3** (**3a** and **3b**) were synthesized by electroreductive cyclization in an electrochemical flow microreactor over 54 min 35 s. The reductive cyclization was carried out by introducing a solution ($n$$Bu_4N\cdot ClO_4$ in THF) containing 0.06 M benzylideneaniline (**1**), 0.12 M terminal dihaloalkane **2** (**2a** and **2d**) and 0.06 M DBU into the electrochemical flow microreactor from a syringe pump under electrolytic conditions (charge passed, 2.15 F mol^{-1}

current density, 38.1 mA cm^{-2}; flow rate, 11 mL h^{-1}; residence time, 3.9 s; collection volume and time for the reaction solution, 10 mL, 54 min 35 s). After collecting the reaction solution, the solvent was removed from the reaction mixture under reduced pressure and filtered through Celite with dichloromethane. The residue was purified by column chromatography using silica gel (hexane/Et_2O/triethylamine = 95/5/0.1%) to afford the products **3a** and **3b**. However, in the synthesis of **3c** by this method, no NMR signals corresponding to **3c** were observed for the reaction mixture; therefore, **3c** could not be isolated.

1,2-Diphenylpiperidine (**3a**):[2] yellow liquid, 78.4 mg, 55% yield. ^{1}H NMR (500 MHz; $CDCl_3$): δ 7.29-7.21 (m, 4H), 7.17-7.11 (m, 3H), 6.89 (d, *J*=8.0 Hz, 2H), 6.75 (t, *J* = 7.2 Hz, 1H), 4.50 (dd, *J* = 6.9, 4.6 Hz, 1H), 3.43-3.38 (m, 1H), 3.29-3.23 (m, 1H), 2.03-1.52 (m, 6H).

1,2-Diphenylpyrrolidine (**3b**):[2] yellow liquid, 75.8 mg, 57% yield. ^{1}H NMR (500 MHz; $CDCl_3$): δ 7.31-7.27 (m, 2H), 7.23-7.18 (m, 3H), 7.15-7.11 (m, 2H), 6.65-6.61 (m, 1H), 6.49 (d, *J* = 8.60 Hz, 2H), 4.72 (d, *J* = 8.00 Hz, 1H), 3.73-3.67 (m, 1H), 3.44-3.37 (m, 1H), 2.42-2.32 (m, 1H), 2.07-1.90 (m, 3H).

2. Fabrication of electrochemical flow microreactor

The detailed procedure for fabrication of the electrochemical flow microreactor is described in the experimental section of the main text.

Figure S1 shows a schematic illustration and a photograph of the electrochemical flow microreactor. Figure S2 shows a schematic illustration of the construction procedure for the electrochemical flow microreactor.

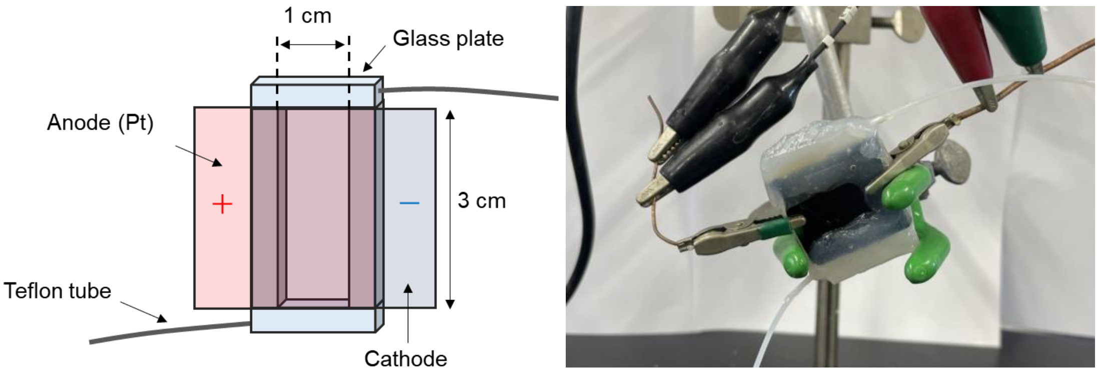

Figure S1: Schematic illustration (adapted with permission from ref. [3]. Copyright 2021 American Chemical Society; this content is not subject to CC BY 4.0) and photograph of the electrochemical flow microreactor.

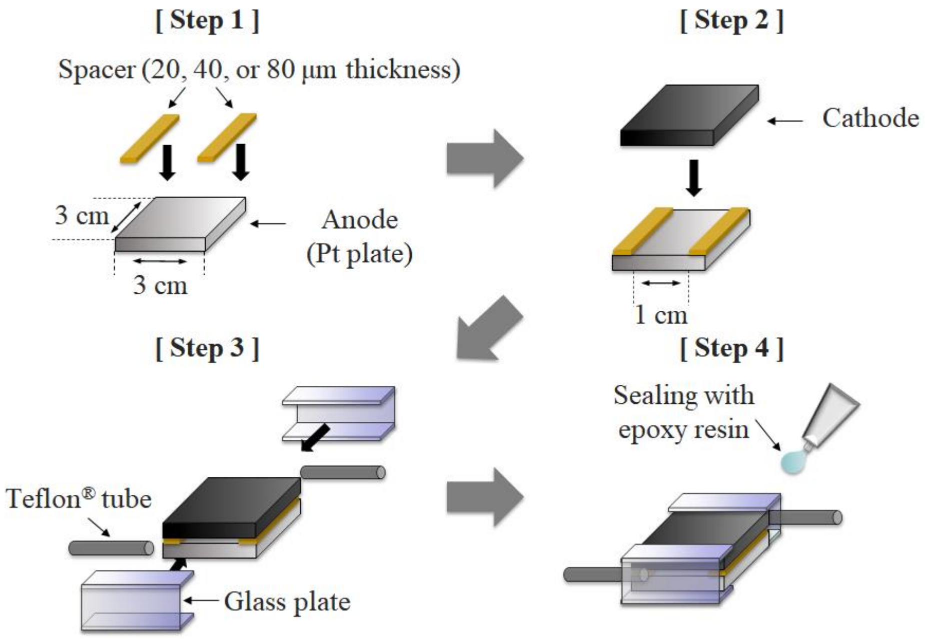

Figure S2: Schematic illustration of procedure for construction of the electrochemical flow microreactor. Adapted with permission from ref. [3]. Copyright 2021 American Chemical Society. This content is not subject to CC BY 4.0.

3. Procedure for electroreductive cyclization in an electrochemical flow microreactor

The detailed procedure for electroreductive cyclization in an electrochemical flow microreactor is described in the experimental section of the main text. Electricity (F mol^{-1}) to the electrolyte as it passes through the reactor was determined by the following equation.

$$\text{Electricity [F mol}^{-1}\text{]} = \frac{\text{Charge passed in microreactor per second [F s}^{-1}\text{]}}{\text{Substrate introduced into microreactor per second [mol s}^{-1}\text{]}}$$

$$= \frac{\frac{1}{96485}\,[\text{F C}^{-1}] \times \left(\text{Current density[mA cm}^{-2}] \times \frac{1}{1000}\,[\text{A mA}^{-1}] \times \text{Electrode surface area[cm}^{2}]\right)\ [\text{C s}^{-1}]}{\left(\text{flow rate[mL h}^{-1}] \times \frac{1}{1000}\,[\text{L mL}^{-1}] \times \frac{1}{3600}\,[\text{h s}^{-1}] \times \text{Concentration of substrate[mol L}^{-1}]\right)\ [\text{mol s}^{-1}]}$$

Figure S3 shows a photograph of the setup for electroreductive cyclization using an electrochemical flow microreactor.

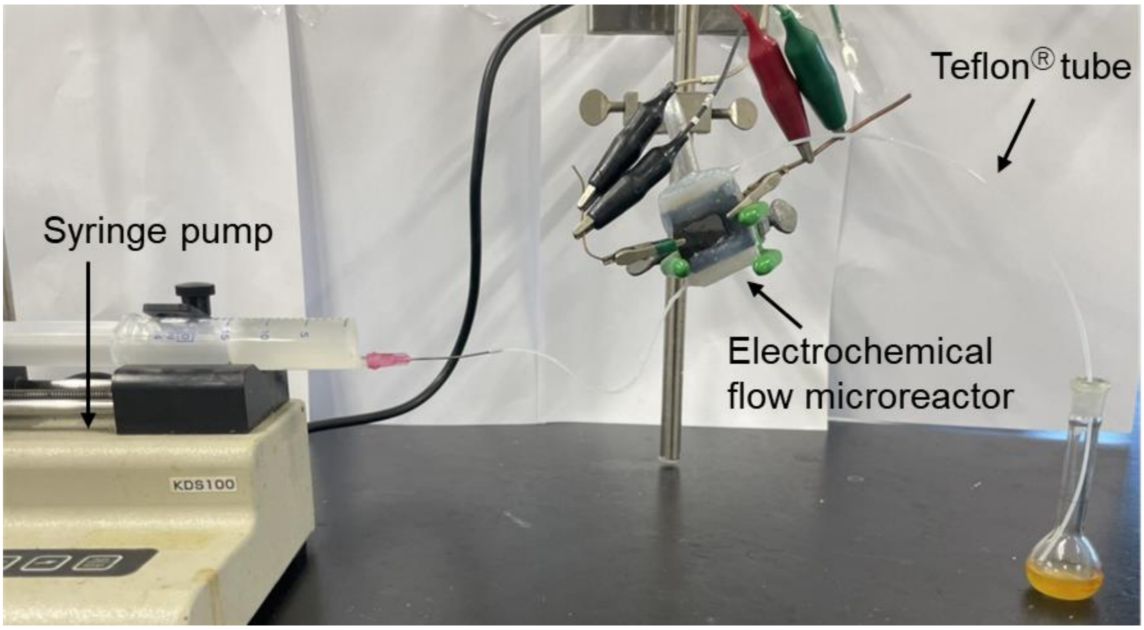

Figure S3: Photograph of the setup for the electroreductive cyclization using an electrochemical flow microreactor.

4. Procedure for electroreductive cyclization in a batch-type reactor

The detailed procedure for electroreductive cyclization in an electrochemical flow microreactor is described in the experimental section of the main text. Figure S4 shows a photograph of the setup for electroreductive cyclization using a batch-type reactor.

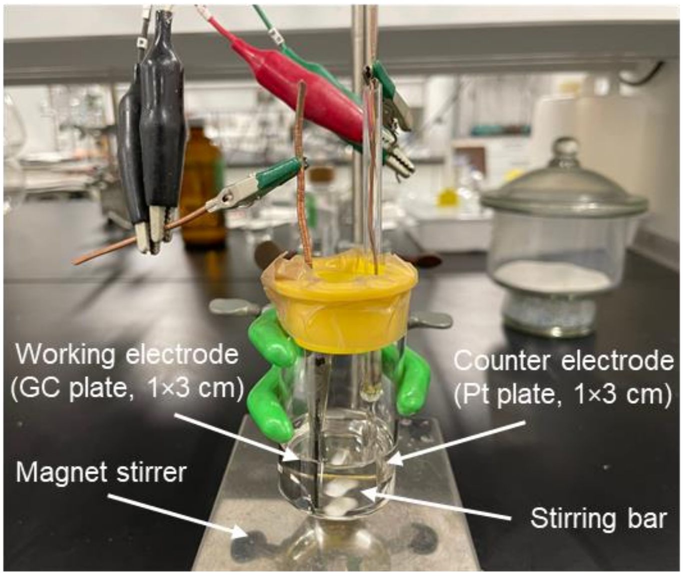

Figure S4: Photograph of the setup for the electroreductive cyclization using a batch-type reactor.

5. Linear sweep voltammetry

5-1. Effect of the electrode material on the reduction potential

LSV measurements were performed to estimate the reduction potentials of benzylideneaniline (**1**) and 1,4-dibromobutane (**2a**) using each of the three electrode materials (Pt, GC, and Ag). 20 mL of 0.1 M *n*$Bu_4N \cdot ClO_4$/THF solution containing 0.06 M benzylideneaniline (**1**) or 0.06 M 1,4-dibromobutane (**2a**), or their mixture was added to a beaker type cell for LSV measurements. N_2 gas was purged through the electrolyte solution with stirring for at least 30 min. Voltammograms were recorded using a Pt, GC, or Ag disk electrode (3.0 mm diameter) at a scan rate of 50 mV s^{-1}. Platinum mesh and Ag/AgCl were used as the counter and reference electrodes, respectively.

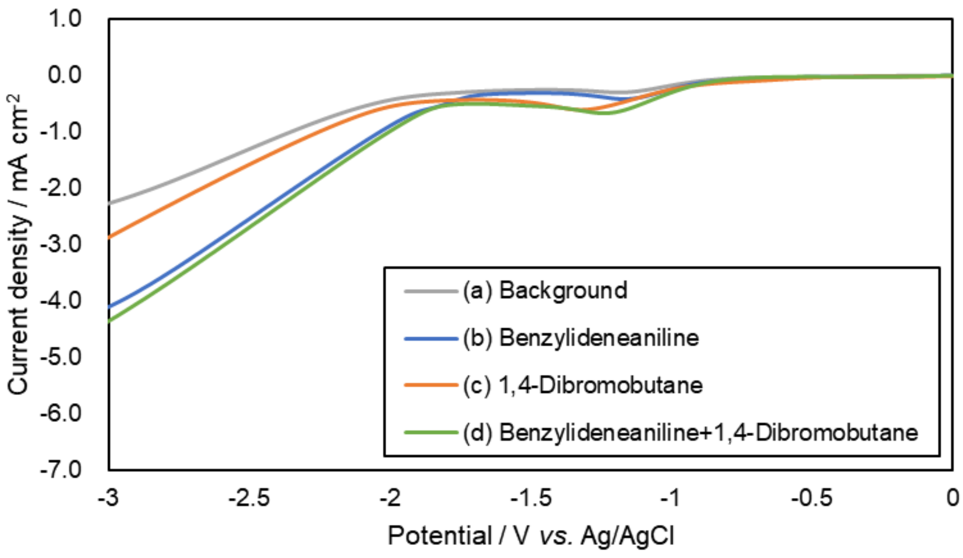

Figure S5: Linear sweep voltammograms of (a) background, (b) 0.06 M benzylideneaniline (**1**), (c) 0.06 M 1,4-diburomobutane (**2a**), and (d) 0.06 M benzylideneaniline (**1**) and 0.06 M 1,4-dibromobutane (**2a**) using a Pt disk electrode.

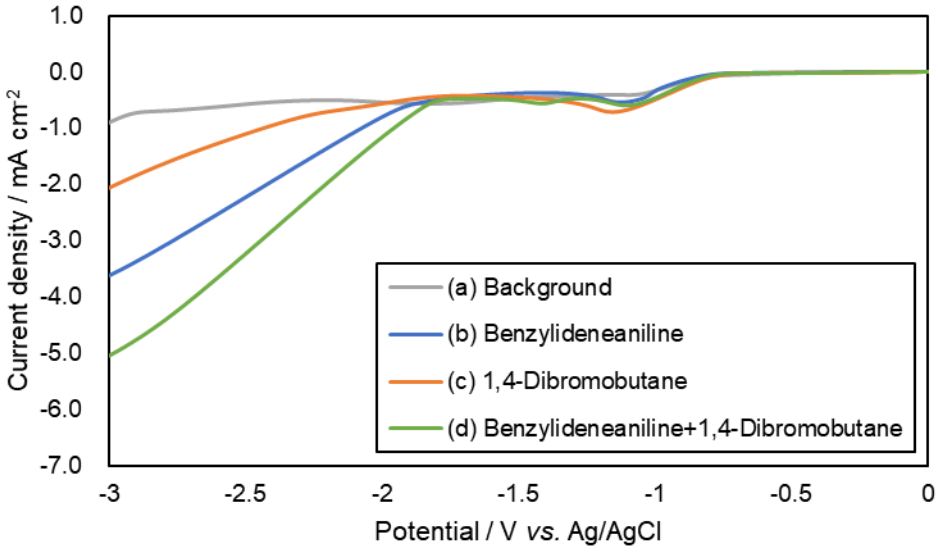

Figure S6: Linear sweep voltammograms of (a) background, (b) 0.06 M benzylideneaniline (**1**), (c) 0.06 M 1,4-diburomobutane (**2a**), and (d) 0.06 M benzylideneaniline (**1**) and 0.06 M 1,4-dibromobutane (**2a**) using a GC disk electrode.

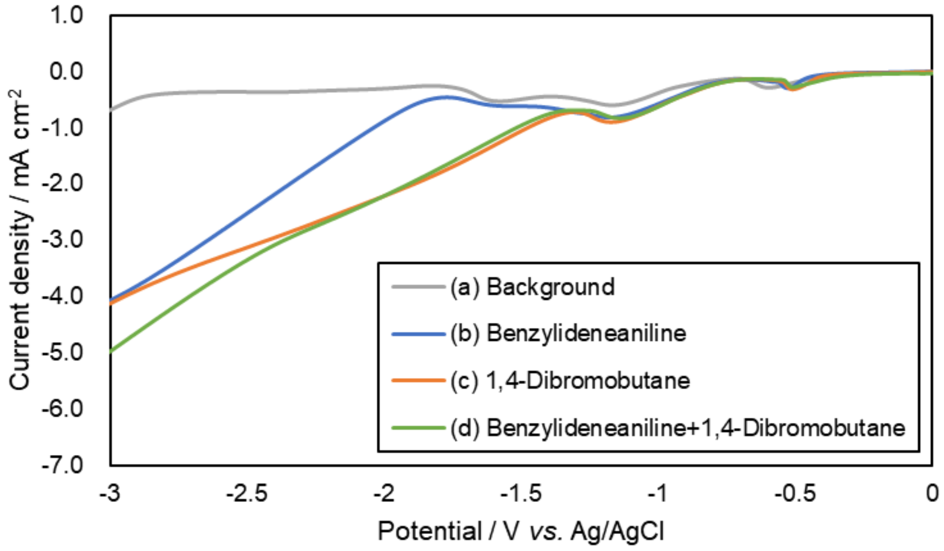

Figure S7: Linear sweep voltammograms of (a) background, (b) 0.06 M benzylideneaniline (**1**), (c) 0.06 M 1,4-diburomobutane (**2a**), and (d) 0.06 M benzylideneaniline (**1**) and 0.06 M 1,4-dibromobutane (**2a**) using a Ag disk electrode.

7-2. Investigation of the reduction potential of various terminal dihaloalkanes

LSV measurements were performed to estimate the reduction potentials of benzylideneaniline **1** and various terminal dihaloalkanes **2** (**2a**–**c**) using a GC disk electrode. 20 mL of 0.1 M *n*$Bu_4N{\cdot}ClO_4$/THF solution containing 0.06 M benzylideneaniline **1** or 0.06 M terminal dihaloalkane **2**, or their mixture was added to a beaker type cell for LSV measurements. N_2 gas was purged through the electrolyte solution with stirring for at least 30 min. Voltammograms were recorded using a GC disk electrode (3.0 mm diameter) at a scan rate of 50 mV s^{-1}. Platinum mesh and Ag/AgCl were used as the counter and reference electrodes, respectively.

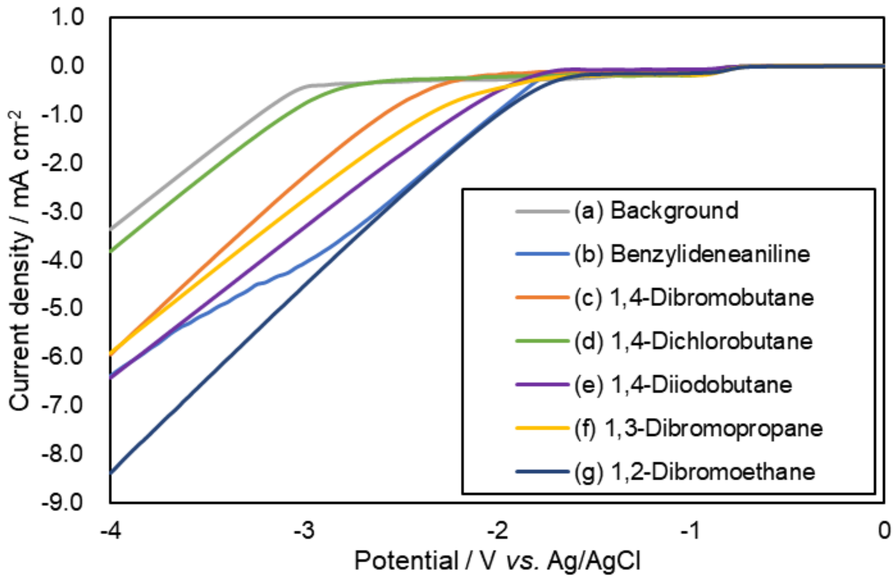

Figure S8: Linear sweep voltammograms of (a) background, (b) 0.06 M benzylideneaniline (**1**), (c) 0.12 M 1,4-dibromobutane (**2a**), (d) 0.12 M 1,4-dichlorobutane (**2c**), (e) 0.12 M 1,4-diiodobutane (**2c**), (f) 0.12 M 1,3-dibromopropane (**2d**), and (g) 0.12 M 1,2-dibromoethane (**2e**).

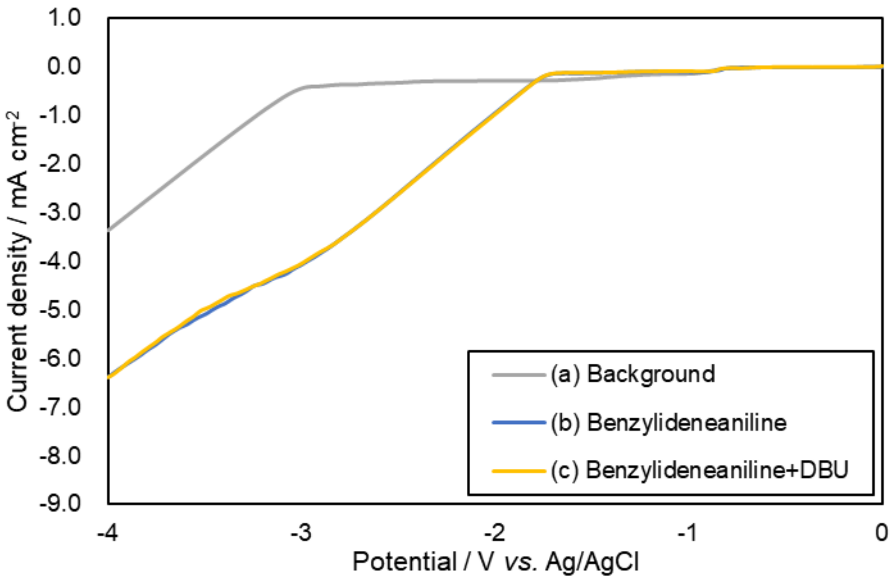

Figure S9: Linear sweep voltammograms of (a) background, (b) 0.06 M benzylideneaniline (**1**), and (c) 0.06 M benzylideneaniline (**1**) and 0.06 M DBU.

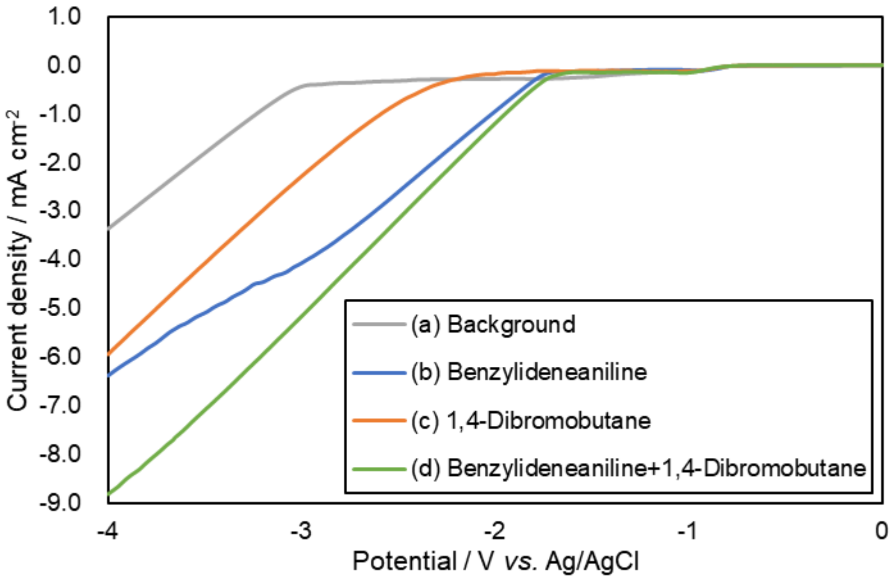

Figure S10: Linear sweep voltammograms of (a) background, (b) 0.06 M benzylideneaniline (**1**), (c) 0.12 M 1,4-dibromobutane (**2a**), and (d) 0.06 M benzylideneaniline (**1**), 0.12 M 1,4-dibromobutane (**2a**), and 0.06 M DBU.

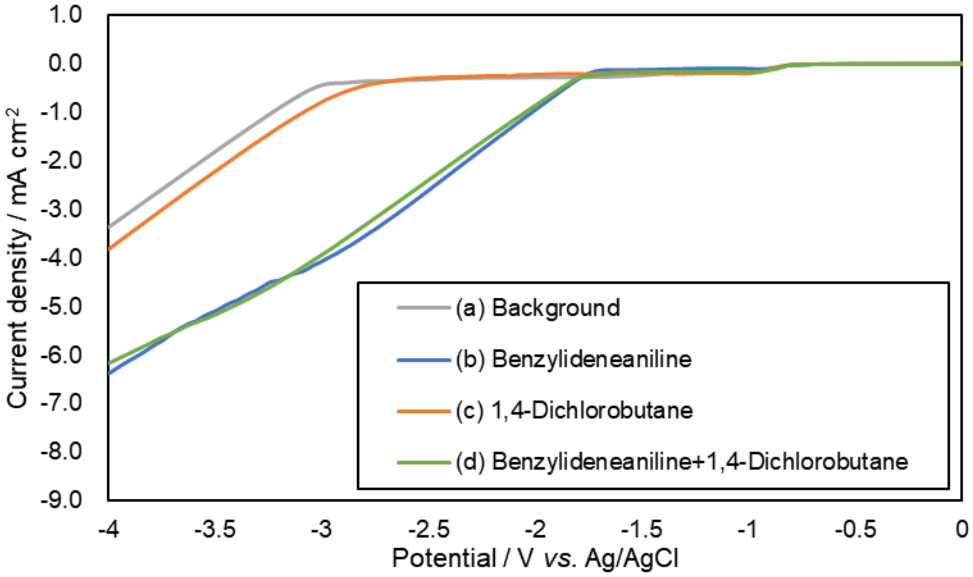

Figure S11: Linear sweep voltammograms of (a) background, (b) 0.06 M benzylideneaniline (**1**), (c) 0.12 M 1,4-dichlorobutane (**2b**), and (d) 0.06 M benzylideneaniline (**1**), 0.12 M 1,4-dichlorobutane (**2b**), and 0.06 M DBU.

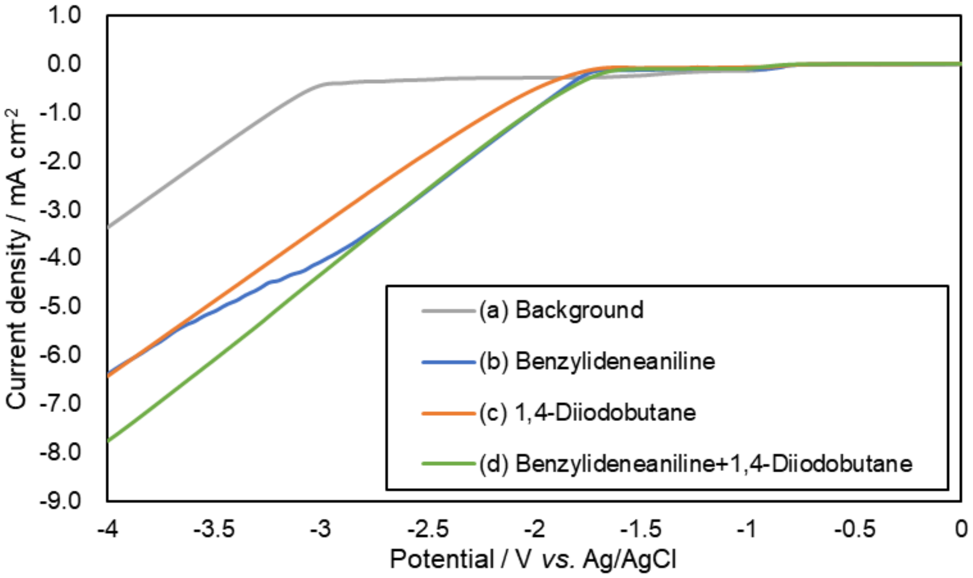

Figure S12: Linear sweep voltammograms of (a) background, (b) 0.06 M benzylideneaniline (**1**), (c) 0.12 M 1,4-diiodobutane (**2c**), and (d) 0.06 M benzylideneaniline (**1**), 0.12 M 1,4-diiodobutane (**2c**), and 0.06 M DBU.

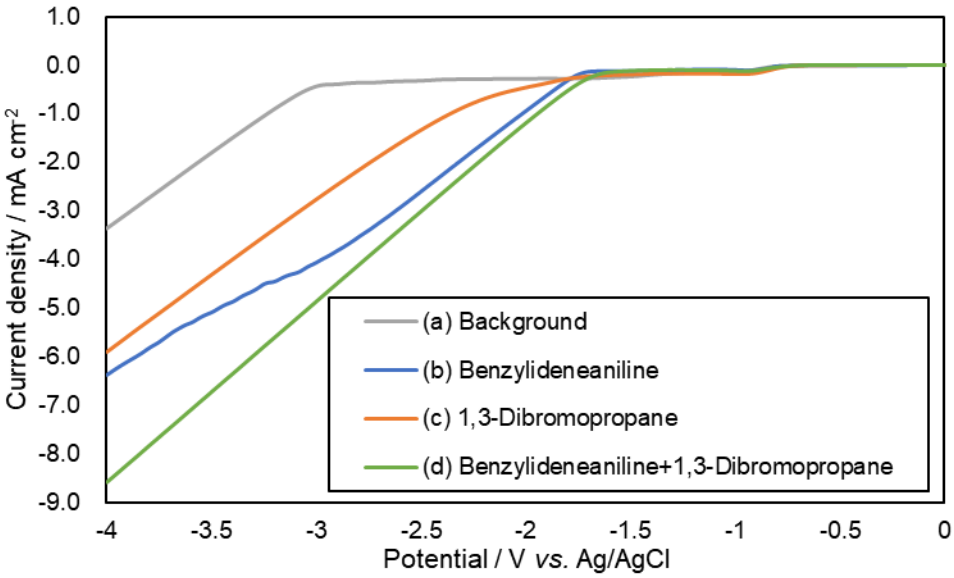

Figure S13: Linear sweep voltammograms of (a) background, (b) 0.06 M benzylideneaniline (**1**), (c) 0.12 M 1,3-dibromopropane (**2d**), and (d) 0.06 M benzylideneaniline (**1**), 0.12 M 1,3-dibromopropane (**2d**), and 0.06 M DBU.

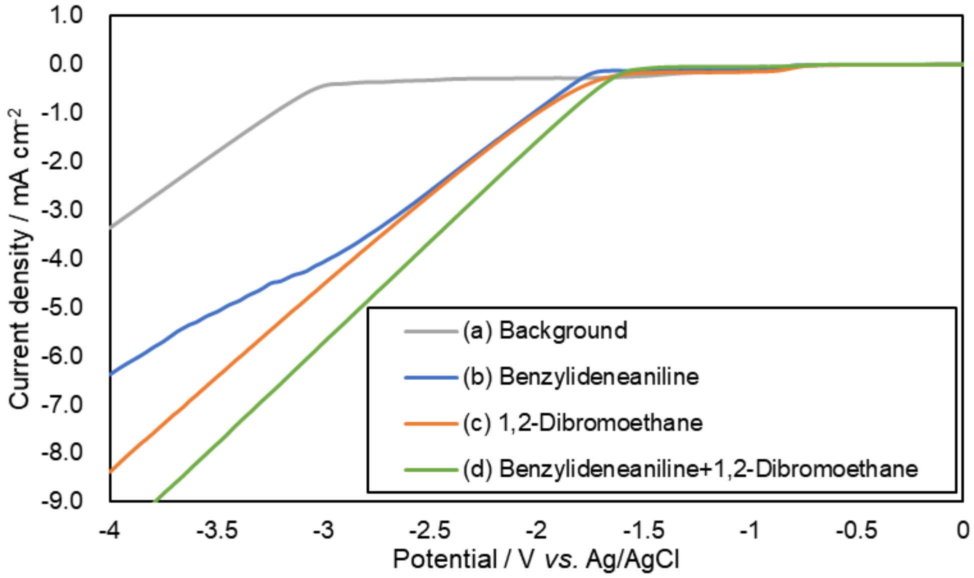

Figure S14: Linear sweep voltammograms of (a) background, (b) 0.06 M benzylideneaniline (**1**), (c) 0.12 M 1,2-dibromoethane (**2e**), and (d) 0.06 M benzylideneaniline (**1**), 0.12 M 1,2-dibromoethane (**2e**), and 0.06 M DBU.

6. HPLC analysis

Typical HPLC chart for the analysis of an electrolyzed solution and calibration curves for HPLC analysis are shown in Figure S15.

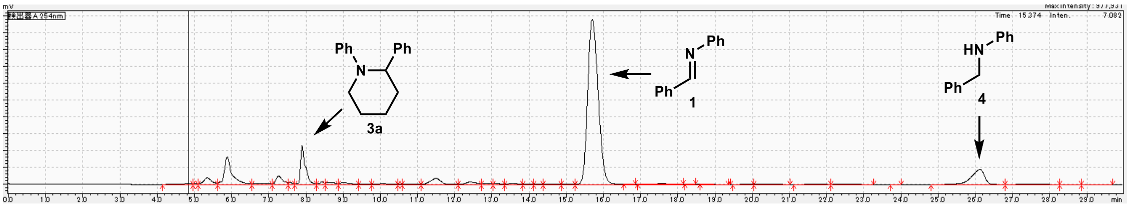

Figure S15: HPLC chart of the reaction mixture after electroreductive cyclization (corresponding to Entry 1 of Table 6).

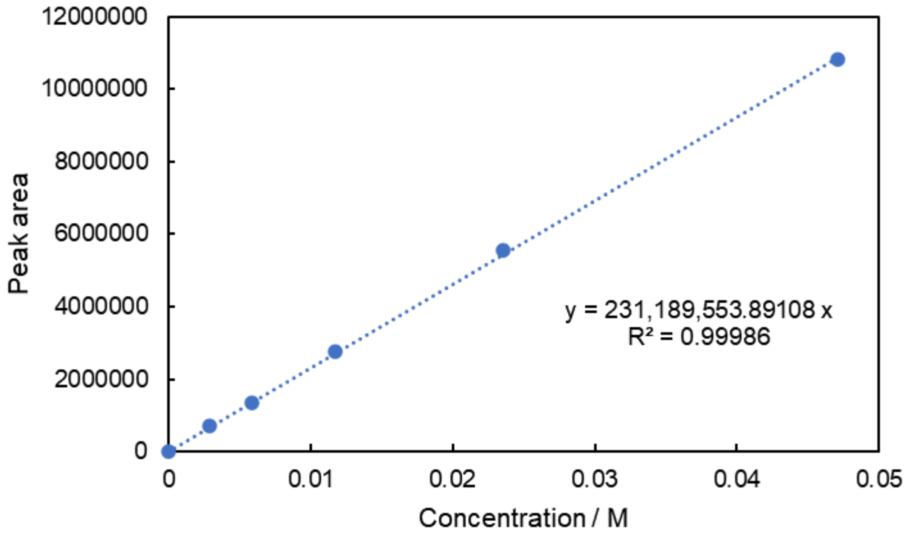

Figure S16: Calibration curve of **3a** for HPLC analysis.

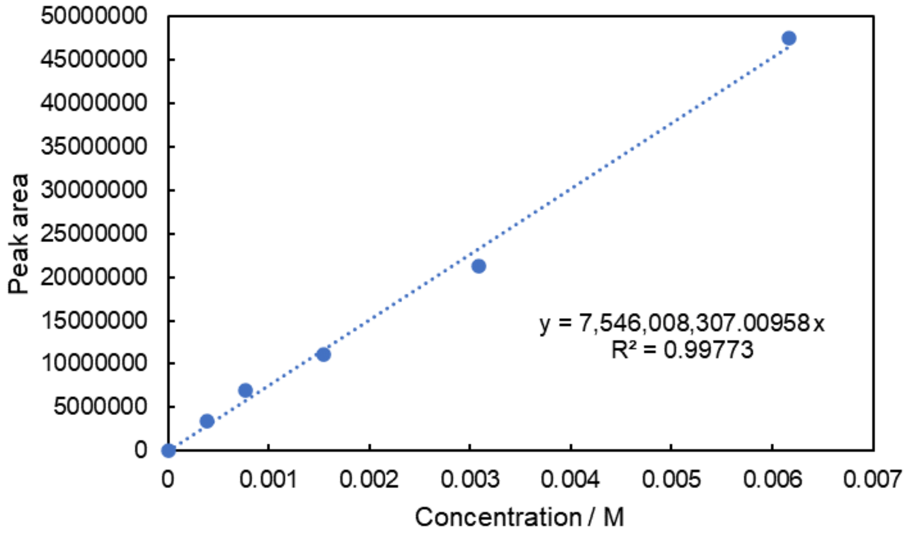

Figure S17: Calibration curve of **1** for HPLC analysis.

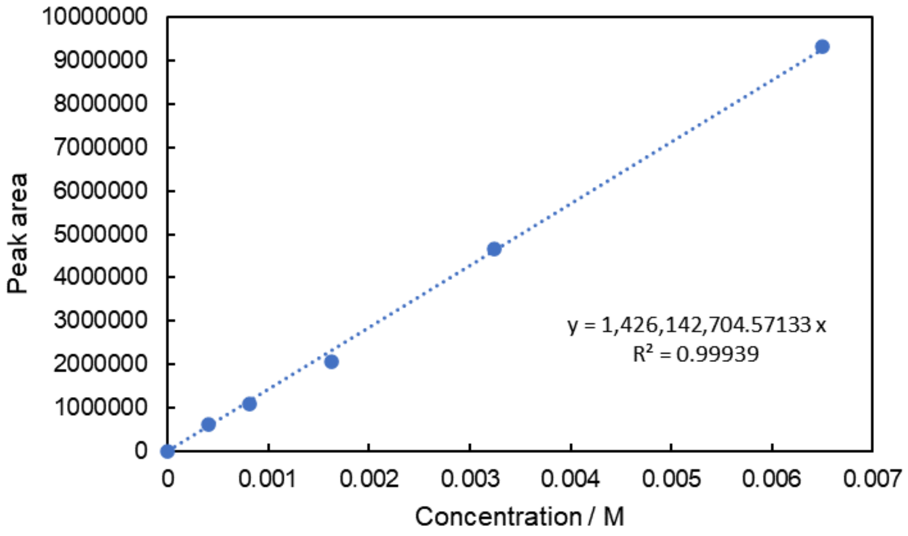

Figure S18: Calibration curve of **4** for HPLC analysis.

7. ¹H NMR spectra

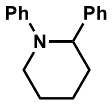

3a

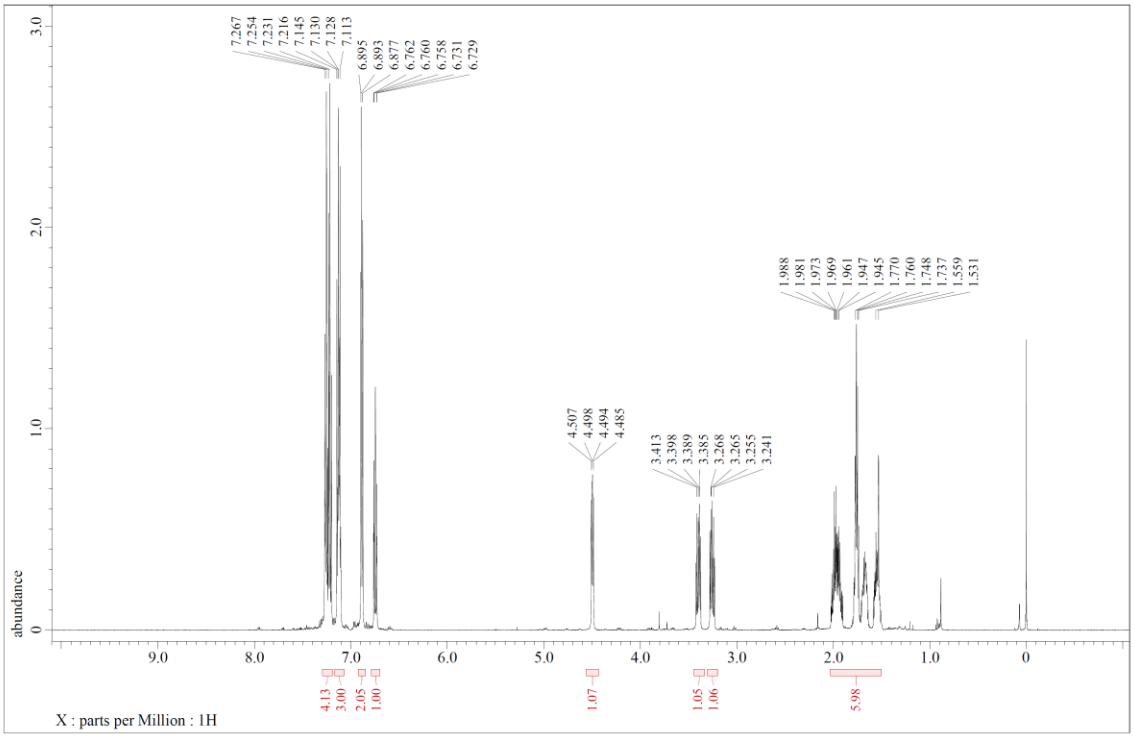

Figure S19: ^{1}H NMR (500 MHz; $CDCl_3$) spectrum of 1,2-diphenylpiperidine (**3a**) synthesized as an authentic sample for HPLC analysis.

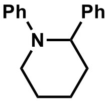

3a

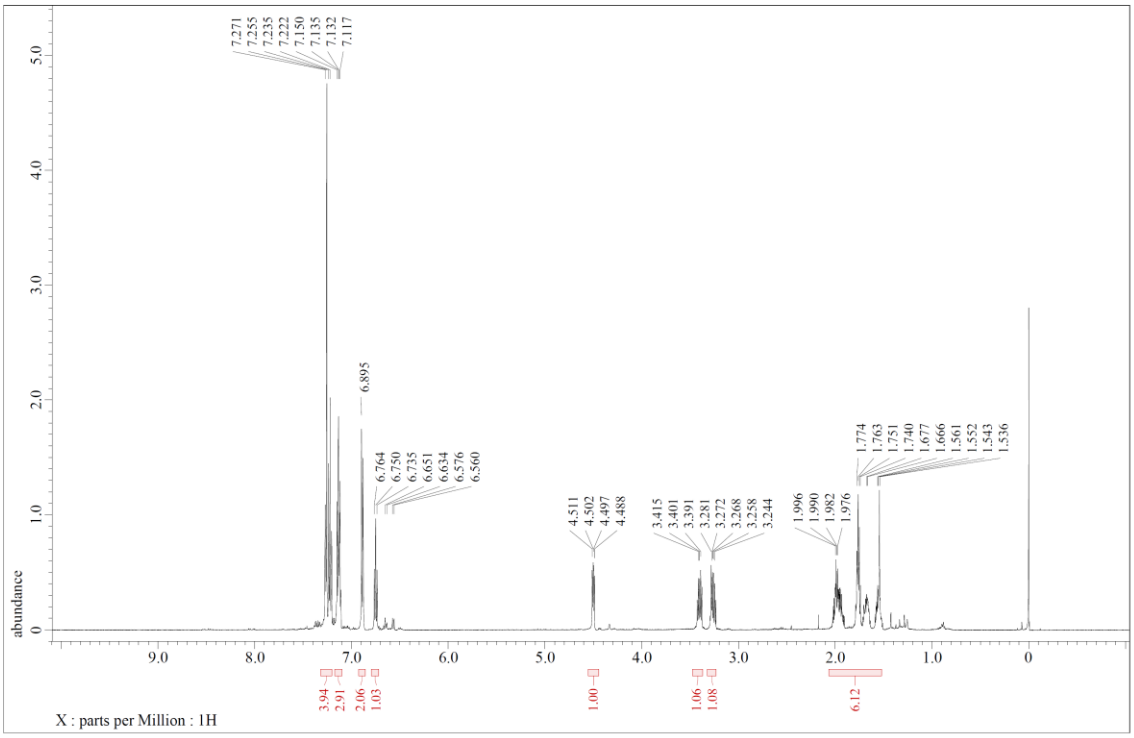

Figure S20: ^{1}H NMR (500 MHz; $CDCl_3$) spectrum of 1,2-diphenylpiperidine (**3a**) synthesized by electroreductive cyclization using an electrochemical flow microreactor.

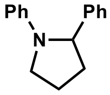

3b

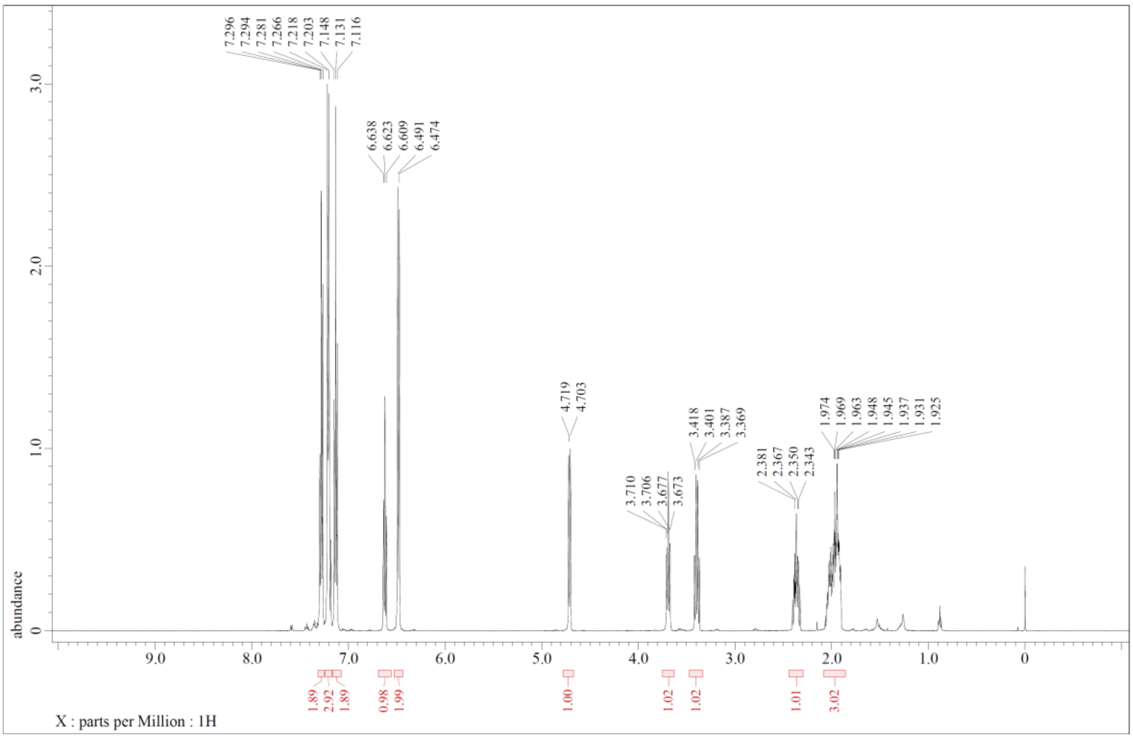

Figure S21: ^{1}H NMR (500 MHz; $CDCl_3$) spectrum of 1,2-diphenylpyrridine (**3b**) synthesized by electroreductive cyclization using an electrochemical flow microreactor.

Supporting references

1 M. A. Topchiy, A. F. Asachenko and M. S. Nechaev, *European J. Org. Chem.*, 2014, **2014**, 3319–3322.

2 D. Wei, C. Netkaew and C. Darcel, *Adv. Synth. Catal.*, 2019, **361**, 1781–1786.

3 Y. Naito, Y. Nakamura, N. Shida, H. Senboku, K. Tanaka and M. Atobe, *J. Org. Chem.*, 2021, **86**, 15953–15960.
